# The Presence of VEGF Receptors on the Luminal Surface of Endothelial Cells Affects VEGF Distribution and VEGF Signaling

**DOI:** 10.1371/journal.pcbi.1000622

**Published:** 2009-12-24

**Authors:** Marianne O. Stefanini, Florence T. H. Wu, Feilim Mac Gabhann, Aleksander S. Popel

**Affiliations:** 1 Department of Biomedical Engineering, Johns Hopkins University School of Medicine, Baltimore, Maryland, United States of America; 2 Department of Biomedical Engineering and Robert M. Berne Cardiovascular Research Center, University of Virginia, Charlottesville, Virginia, United States of America; Charité - Universitätsmedizin Berlin, Germany

## Abstract

Vascular endothelial growth factor (VEGF) is a potent cytokine that binds to specific receptors on the endothelial cells lining blood vessels. The signaling cascade triggered eventually leads to the formation of new capillaries, a process called angiogenesis. Distributions of VEGF receptors and VEGF ligands are therefore crucial determinants of angiogenic events and, to our knowledge, no quantification of abluminal vs. luminal receptors has been performed. We formulate a molecular-based compartment model to investigate the VEGF distribution in blood and tissue in humans and show that such quantification would lead to new insights on angiogenesis and VEGF-dependent diseases. Our multiscale model includes two major isoforms of VEGF (VEGF_121_ and VEGF_165_), as well as their receptors (VEGFR1 and VEGFR2) and the non-signaling co-receptor neuropilin-1 (NRP1). VEGF can be transported between tissue and blood via transendothelial permeability and the lymphatics. VEGF receptors are located on both the luminal and abluminal sides of the endothelial cells. In this study, we analyze the effects of the VEGF receptor localization on the endothelial cells as well as of the lymphatic transport. We show that the VEGF distribution is affected by the luminal receptor density. We predict that the receptor signaling occurs mostly on the abluminal endothelial surface, assuming that VEGF is secreted by parenchymal cells. However, for a low abluminal but high luminal receptor density, VEGF binds predominantly to VEGFR1 on the abluminal surface and VEGFR2 on the luminal surface. Such findings would be pertinent to pathological conditions and therapies related to VEGF receptor imbalance and overexpression on the endothelial cells and will hopefully encourage experimental receptor quantification for both luminal and abluminal surfaces on endothelial cells.

## Author Summary

Angiogenesis is the growth of new blood vessels from pre-existing vasculature that occurs in physiological (e.g., exercise) and pathological contexts (e.g., cancer). This process is often triggered by a signaling cascade that occurs upon ligand-receptor binding between vascular endothelial growth factor (VEGF) and its receptors (VEGFR1/Flt-1, VEGFR2/KDR). These receptors are expressed by endothelial cells that line the blood vessels. Little is known about the quantitative proportion of abluminal receptors (facing the tissue) as compared to those on the luminal surface (facing the blood). We have built a compartment model with molecular details from human tissues to investigate why such experimental data would be of importance. We conclude that the receptor distribution on the endothelial cells can significantly alter the VEGF distribution and the VEGF signaling (through its binding to the receptors) and that quantification of luminal vs. abluminal VEGF receptors would shed light on VEGF signaling and VEGF-dependent mechanisms of angiogenesis.

## Introduction

Physiologic angiogenesis, the growth of new capillaries from pre-existing blood vessels, occurs in wound healing, pregnancy, exercise, and embryonic development. Diseases such as cancer and age-related macular degeneration are angiogenesis-dependent [1].

The growth of new capillaries from pre-existing blood vessels is mediated by several growth factors, one of which is a potent family of cytokines called vascular endothelial growth factor (VEGF). The VEGF family is composed of five members: VEGF-A (often referred to as VEGF), VEGF-B, VEGF-C, VEGF-D and placental growth factor (PlGF). Alternative splicing of VEGF-A provides about 13 different VEGF isoforms [2],[3]. Human VEGF consists of at least seven isoforms: VEGF_121_, VEGF_145_, VEGF_148_, VEGF_165_, VEGF_183_, VEGF_189_, and VEGF_206_ [4],[5]. Although VEGF_121_, VEGF_165_, VEGF_183_ are diffusible, VEGF_189_ and VEGF_206_ are mainly sequestered in the extracellular matrix [4]. Amongst the major isoforms (with length 121, 165, 189 and 206 amino acids), VEGF_121_ and VEGF_165_ are more highly expressed than VEGF_189_ and VEGF_206_. Furthermore, the roles of VEGF_189_ and VEGF_206_ in vivo remain to be clearly identified [3]. For these reasons, we only consider VEGF_121_ and VEGF_165_ isoforms in the present model. These two isoforms bind VEGF receptors, VEGFR1 (fms-related tyrosine kinase 1 or Flt-1 in humans) and VEGFR2 (kinase insert domain receptor also designated as Flk-1, or KDR in humans). VEGF_165_ binds to the non-signaling co-receptor neuropilin-1 (NRP1) as well and serves as a bridge for the VEGFR2-NRP1 complex. It has been shown recently that VEGF_121_ may also bind to NRP1; however, this binding is not sufficient to bridge the VEGFR2-NRP1 complex [6]. Preliminary sensitivity analyses from our group suggest that incorporation of the binding between VEGF_121_ and NRP1 does not drastically change the predictions regarding the VEGF distribution [7]. Therefore, this binding is not included in the model at the moment; this can be modified when more information becomes available. Finally, VEGF_165_ contains a heparin binding domain, which allows it to bind to the heparan sulfate glycosaminoglycan (GAG) chains of the extracellular matrix and the cellular basement membranes [8].

We have introduced a compartment model of VEGF distribution in the human body [9]. In the “healthy” set-up, the system was composed of two main compartments: the blood (vascular system) and the rest of the body. A third compartment was added for pathological cases to distinguish the diseased from the healthy tissue. VEGF_121_, VEGF_165_, and their respective interactions with VEGFR1, VEGFR2 and NRP1 were considered. VEGF was secreted by the parenchymal cells (in the healthy tissue) and the tumor cells (when the diseased tissue was assumed to be a breast cancer tumor). Other elements in the blood, such as platelets and granulocytes, sequester large amounts of VEGF and could potentially release significant amounts of VEGF as well [10]; the role of these processes in VEGF balance in the body is not known. However, since the rates of VEGF release from these blood elements have not been quantified, we have decided, as a first approximation, to neglect explicit representation of these sources; a distinct mathematical term can be added to the equations to model VEGF release from these elements in the future. We assume that the compartments are well-mixed and that freely diffusible (unbound) VEGF is transported by vascular permeability between the tissues and the blood.

The model presented here is an extension of our previously published model [9], as was a recent study that analyzed the effects of soluble VEGFR1 [7],[11]. Two major additions were made. First, lymphatic drainage of VEGF was added, serving as a second route for VEGF to be transported from the tissue to the blood compartment. Secondly, our previous model considered VEGF receptors to be solely expressed on the abluminal endothelial surface. Here, we included the presence of VEGF receptors and co-receptor NRP1 on the luminal endothelial surface based on the evidence that VEGFR2 (Flk-1, KDR) is also present on the luminal endothelial surface [12].

We hypothesize that the distribution of VEGF receptors between the abluminal and luminal surfaces of the endothelial cells (i.e., present solely on the abluminal endothelial surface; present solely on the luminal endothelial surface; or present on both surfaces of the endothelial cells) can impact the VEGF ligand distribution in the tissue and in the blood, as well as the VEGF signaling efficiency. The focus of this paper is to investigate the effects of receptor repartition on endothelial cellular surfaces and emphasize the importance of receptor quantification.

## Materials and Methods

### 

#### Geometry

**Figure 1:**
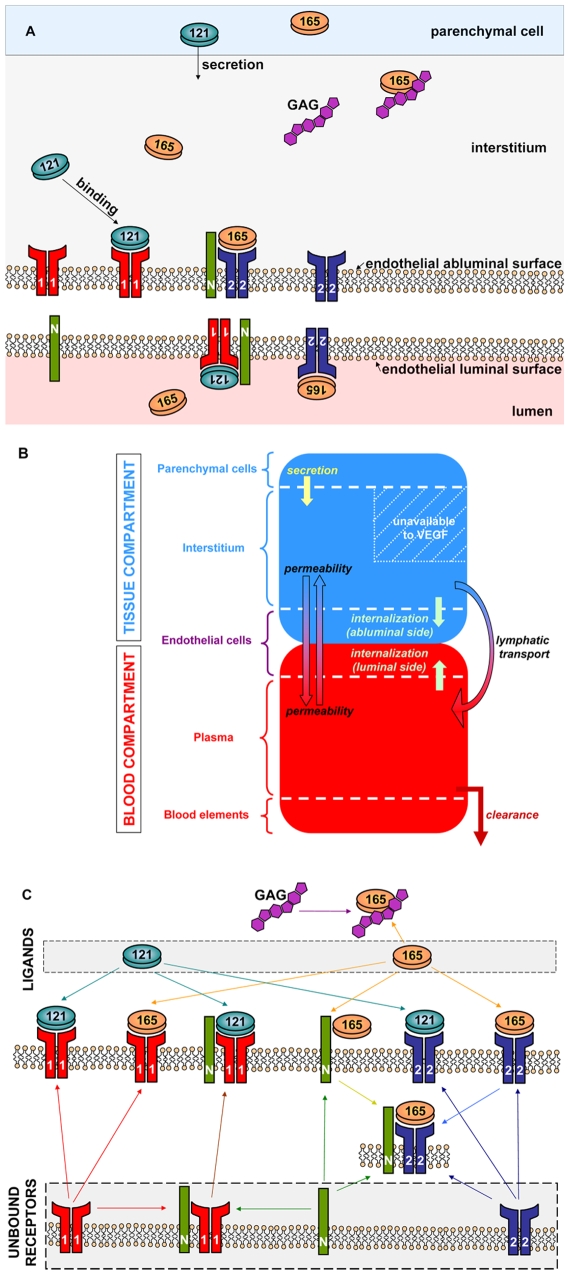
Compartment model. A. Schematic of a tissue cross section. B. Compartment model. The vascular system is separated from the rest of the body. The blood compartment comprises the plasma (available to VEGF), the blood elements (blood cells, fibrin, clotting elements, etc.), as well as the luminal side of the basement membranes of the endothelial cells. The tissue compartment is composed of the parenchymal cells that secrete VEGF, the interstitium as well as the abluminal surface of the endothelial cells lining the capillaries. The fraction that is not accessible to VEGF is represented as a hatched area. The arrows illustrate inter-compartment and intra-compartment exchanges: secretion, vascular permeability, internalization of the receptors, lymphatic drainage, and clearance from the plasma. C. Schematic of the chemical interactions. Two isoforms of VEGF are considered: VEGF_121_ and VEGF_165_. Free receptors (VEGFR1, VEGFR2 and NRP1) and free VEGF isoforms are located in the gray areas. VEGF_121_ and VEGF_165_ both bind VEGFR1 and VEGFR2. VEGF_165_ also binds glycosaminoglycan chains (GAG) as well as the co-receptor NRP1. VEGF_165_ can serve as a bridge for the formation of VEGFR2-NRP1 complex. Finally, VEGF_121_ can bind to VEGFR1-NRP1 complex.

The model has been fully described in our previous paper [9]. To summarize, we distinguish between the vascular system (blood) and the rest of the body (represented by skeletal muscle). The tissue is divided into parenchymal cells and capillaries, separated by the interstitial space. This space is further subdivided into the extracellular matrix (ECM) and the basement membranes of the parenchymal cells and of the endothelial cells (PBM and EBM respectively). Secreted by parenchymal cells in the tissue, VEGF isoforms VEGF_121_ and VEGF_165_ diffuse freely and bind to VEGF receptors and neuropilin-1 that are expressed on the endothelial surfaces, as shown in Figure 1A.

#### Computational model

A schematic of the computational design is illustrated in Figure 1B. We distinguish between the vascular system (“blood compartment”) and the tissue (“tissue compartment”). The tissue compartment is divided into two parts: the parenchymal cells where VEGF is secreted, and the interstitium. However, because the extracellular matrix is a porous medium, and because some pores are not accessible to freely diffusible molecules, only a fraction of the interstitial space is accessible to VEGF. This accessible region, called available fluid volume *U_AV_*, is to be distinguished from the rest of the interstitial space. It is possible to link the available fluid volume to the total volume of the tissue *U* by the relation *U_AV_ = K_AV_. U*, where *K_AV_* represents the ratio between the available fluid interstitial space to the total volume, and can be expressed as the product of the partition coefficient and the porosity of the medium. Similarly, we partition the blood into plasma and the rest of the blood. Further details about the available fluid volumes for VEGF can be found in our previous study [9].

The model includes the expression of receptors on the luminal and abluminal endothelial surfaces as well as their internalization. In the mathematical setup, the receptors expressed on the abluminal endothelial surface are considered to be part of the “tissue compartment” while the receptors expressed on the luminal endothelial surface are part of the “blood compartment.” This permits a clear distinction between the two surfaces of the endothelial cells and their receptor expressions as illustrated in Figure 1B.

Inter-compartment transport modes include vascular permeability and lymphatic removal. VEGF can extravasate and intravasate (bi-directional microvascular permeability). Note that hemodynamics is not considered in this compartment model because there is no evidence that the transport of VEGF is blood flow limited. The cytokine can also be drained from the tissue into the blood (unidirectional lymphatic drainage) or cleared from the plasma (e.g., by the kidneys or the liver, organs that are not explicitly represented in the model).

Figure 1C summarizes the biochemical reactions that are included in our model. We consider two VEGF isoforms: VEGF_121_ and VEGF_165_. Both isoforms bind to two receptor tyrosine kinases: VEGFR1 and VEGFR2. VEGF-VEGFR complex formation induces signal transduction in vivo. The model also includes the binding of VEGF_165_ to neuropilin-1 (NRP1). Although VEGF_121_ has been shown to bind to NRP1 as well, this binding does not bridge VEGFR2-NRP1 complex as VEGF_165_ does [6]. The inclusion of such binding to our model does not significantly affect the VEGFR2 signaling pathway, nor does it significantly change the VEGF distribution profile [7]. Therefore, our present model does not include the possibility of VEGF_121_-NRP1 complex formation, but this could be readily added when kinetic information and quantitative data become available. Ternary groups can be formed either by the coupling of NRP1 and VEGFR1 then binding to VEGF_121_ or by VEGF_165_ bridging VEGFR2 and NRP1 to form a VEGFR2-VEGF_165_-NRP1 triplet. Besides binding to VEGFR1, VEGFR2 and NRP1, VEGF_165_ contains a heparin-binding domain that permits the isoform to be sequestered by the extracellular matrix or the cellular basement membranes.

#### Biochemical kinetic equations

The equations as well as a glossary of each term are summarized in Text S1. The concentrations are all expressed in moles/cm^3^ tissue. The equations (S.18) and (S.20) describing the temporal dependence of the free ligand concentrations in the available fluid interstitial space need to be modified to take into account the introduction of lymphatic drainage of VEGF from the tissue to the blood.
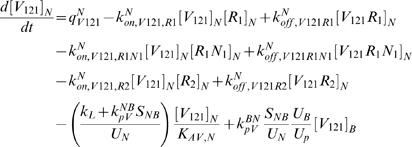
(1)

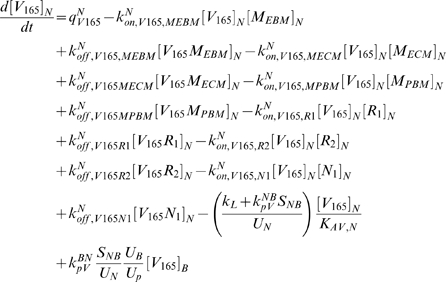
(2)where *k_L_* is the lymph flow rate (in cm^3^/s). The physical meaning of each term is described in Text S1.

In the blood compartment, the introduction of luminal receptors leads to new equations: the equations (S.7) to (S.17) governing the unligated and ligated receptor concentrations in the tissue compartment are now applicable to the blood compartment as well. The introduction of the VEGF lymphatic drainage also changes equations (S.21) and (S.22) describing the temporal dependence of the free ligand concentrations in the plasma. We use *k_L_* to denote the rate of lymphatic flow rate from the tissue to the blood. Equations (S.21) and (S.22) become
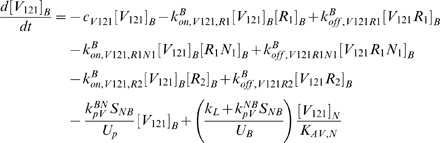
(3)

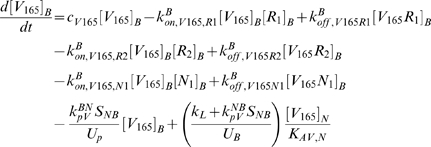
(4)


#### Numerical implementation

The system is described by 32 ordinary non-linear differential equations (19 for the tissue compartment and 13 for the blood compartment). These equations and the initial conditions were implemented using Visual FORTRAN 6 software on a PC. Transient solutions were calculated using an adaptive step-size Runge-Kutta 5^th^-order accuracy integrative scheme. A relative error tolerance of 10^−5^ was used. The steady state was defined when the concentrations changed by less than 1%.

#### Model parameters

The parameters are summarized in Tables 1–5. We model a 70-kg human subject. This includes 5.154 liters of total blood, of which 54.3% constitutes the blood plasma (2.717 liters).

**Table 1:** Geometric parameters for the tissue (human vastus lateralis muscle).

Skeletal muscle characteristic	Parameter	Value	Unit	Ref
***Muscle fibers***	Cross-sectional area of one fiber	3000	µm^2^	[18]
	Perimeter of one fiber	222	µm	[18]
***Capillary-fiber ratio***		1.38		[23]
***Capillary density***		420	capillaries/mm^2^ tissue	[18]
***Muscle fiber density***		304	fibers/mm^2^ tissue	[18]
***Volume fractions***	Interstitial space	8.16%	cm^3^/cm^3^ tissue	[24],[25]
	Fibers	89.98%	cm^3^/cm^3^ tissue	[18]
	Microvessels	1.86%	cm^3^/cm^3^ tissue	[18]
	of which vascular space	1.4%	cm^3^/cm^3^ tissue	[26]
***Microvessels***	Internal diameter of microvessel	6.56	µm	[18]
	Thickness of endothelial cell	0.5	µm	[27]
	External diameter of microvessel	7.56	µm	[18]
	Cross-sectional area of one microvessel	45	µm^2^	[18]
	Perimeter of one microvessel	26	µm	[18]
***Surface areas***	Muscle fibers	664	cm^2^/cm^3^ tissue	[18]
	Microvessels	108	cm^2^/cm^3^ tissue	[18]
***Basement membranes (BM)***	Thickness of muscle fiber BM	24	nm	[28]
	Basement membrane volume (muscle fiber)	0.00159	cm^3^/cm^3^ tissue	[9]
	of which available to VEGF	0.000307	cm^3^/cm^3^ tissue	[9]
	Thickness of microvessel BM	43	nm	[28]
	Basement membrane volume (microvessel)	0.00045	cm^3^/cm^3^ tissue	[9]
	of which available to VEGF	0.000087	cm^3^/cm^3^ tissue	[9]
	Extracellular matrix volume	0.07951	cm^3^/cm^3^ tissue	[9]
	of which available to VEGF	0.061987	cm^3^/cm^3^ tissue	[9]
***Skeletal muscle nuclear domain (SMND) surface area***		1850	µm^2^	[18]

**Table 2 pcbi-1000622-t002:** Kinetic parameters of VEGF in the tissue (human vastus lateralis muscle).

Reaction	Parameter	Measured parameter		Tissue Model	
		Value	Unit	Value	Unit
***VEGF binding to VEGFR1***	k_on_	3 10^7^	M^−1^ s^−1^	4.8 10^−1^	(pmol/cm^3^ tissue) ^−1^ s^−1^
	k_off_	10^−3^	s^−1^		
	K_d_	33	pM	2.0 10^−3^	pmol/cm^3^ tissue
***VEGF binding to VEGFR2***	k_on_	10^7^	M^−1^ s^−1^	1.6 10^−1^	(pmol/cm^3^ tissue) ^−1^ s^−1^
	k_off_	10^−3^	s^−1^		
	K_d_	100	pM	6.4 10^−3^	pmol/cm^3^ tissue
***VEGF165 binding to NRP1***	k_on_	3.2 10^6^	M^−1^ s^−1^	5.1 10^−2^	(pmol/cm^3^ tissue) ^−1^ s^−1^
	k_off_	10^−3^	s^−1^		
	K_d_	312	pM	2.0 10^−2^	pmol/cm^3^ tissue
***VEGF165 binding to GAGs***	k_on_	4.2 10^5^	M^−1^ s^−1^	6.7 10^−3^	(pmol/cm^3^ tissue) ^−1^ s^−1^
	k_off_	10^−2^	s^−1^		
	K_d_	24	nM	1.5	pmol/cm^3^ tissue
***Coupling of NRP1 & VEGFR2***	k_cV165R2,N1_	3.1 10^13^	(mol/cm^2^) ^−1^ s^−1^	2.8 10^−1^	(pmol/cm^3^ tissue) ^−1^ s^−1^
	k_offV165R2,N1_	10^−3^	s^−1^		
	k_cV165N1,R2_	10^14^	(mol/cm^2^) ^−1^ s^−1^	9.2 10^−1^	(pmol/cm^3^ tissue) ^−1^ s^−1^
	k_offV165N1,R2_	10^−3^	s^−1^		
***VEGFR1 coupling to NRP1***	k_cR1,N1_	10^14^	(mol/cm^2^) ^−1^ s^−1^	9.2 10^−1^	(pmol/cm^3^ tissue) ^−1^ s^−1^
	k_dissocR1,N_	10^−2^	s^−1^		
***VEGFR internalization***	k_int,R_	2.8 10^−4^	s^−1^		
	k_int,C_	2.8 10^−4^	s^−1^		

In this table, 6.24 10^7^ (pmol/cm^3^ tissue)/M and 1.09 10^14^ (pmol/cm^3^ tissue)/(mol/cm^2^ EC). Here, M = moles/liter available interstitial fluid volume. The derivation of these parameters is found in [18].

**Table 3 pcbi-1000622-t003:** VEGF concentration and receptor densities for the tissue (human vastus lateralis).

Category	Parameter	Measured parameter		Tissue model	
		Value	Unit	Value	Unit
***Free VEGF concentration***	Human vastus lateralis, rest	1	pM	6.2 10^−5^	pmol/cm^3^ tissue
***Total VEGF tissue concentration***	Human vastus lateralis, rest	1–2	pg/µg protein	3.4–6.9	pmol/cm^3^ tissue
***VEGFR1 tissue concentration***	Human vastus lateralis, rest	1.6–1.8	pg/µg protein	1.1–1.2	pmol/cm^3^ tissue
				60,000–68,000	#/EC
***VEGFR2 tissue concentration***	Human vastus lateralis, rest	0.33–0.5	pg/µg protein	0.24–0.34	pmol/cm^3^ tissue
				13,000–19,000	#/EC
***NRP1 tissue concentration***				0.018–1.8	pmol/cm^3^ tissue
				1,000–100,000	#/EC
***ECM binding site density***	ECM	0.75	µM	46	pmol/cm^3^ tissue
	Vessel BM	13	µM	1	pmol/cm^3^ tissue
	Myocyte BM	13	µM	4	pmol/cm^3^ tissue

The conversion of receptor densities to tissue: see table 2. Endothelial cell surface area = 1000 µm^2^. Conversions are as follow: VEGF concentration: 6.2 10^7^ (pmol/cm^3^ tissue)/M (here, M = moles/liter interstitial fluid available to VEGF); VEGF binding sites in the ECM and BMs: 6.2 10^7^ (pmol/cm^3^ tissue)/M (ECM fluid accessible to VEGF), 5.7 10^4^ (pmol/cm^3^ tissue)/M (EBM fluid accessible to VEGF), 3.1 10^5^ (pmol/cm^3^ tissue)/M (MBM fluid accessible to VEGF). For example, M(EBM) = moles/liter endothelial basement membrane. Conversions from pg/mg protein are based on 155 mg protein/g of tissue and 45 kDa VEGF, 210 kDa VEGFR1, 240 kDa VEGFR2 [18].

**Table 4 pcbi-1000622-t004:** Geometric parameters of the compartments [9].

Category	Parameter	Value	Unit
***Tissue compartment***	Total volume	61321	cm^3^
	Available fluid volume for VEGF	3825	cm^3^
***Blood compartment***	Total volume	5	L
	Available fluid volume for VEGF (plasma)	2.717	L

**Table 5:** Kinetic parameters between the compartments and in the blood.

Category	Parameter	Value	Unit	Reference
***Inter-compartment***	Vascular permeability to VEGF *k_p_*	4×10^−8^	cm/s	[9]
	Lymph flow rate *k_L_*	120	mL/h	[15]
		2	cm^3^/min	
***Blood compartment***	Clearance rate for VEGF *c_V_*	0.0648	min^−1^	[16]

The volume of the normal tissue corresponds to that of a 70-kg human subject with a skeletal muscle density of 1.06 g/cm^3^ after subtracting 5,154 cm^3^ of whole blood, i.e., 61,321 cm^3^. The parameters and the properties of the skeletal muscle (tissue compartment) are summarized in Tables 1–4. Briefly, the fluid volume fractions available for VEGF in the extracellular matrix, the parenchymal basement membrane and the endothelial basement membrane are 6.1987%, 0.0307% and 0.0087% of the total tissue respectively (Table 1). Thus, the interstitial fluid volume accessible by VEGF is 6.2381% of the total volume (Table 4). Total VEGF expression isoform ratio VEGF_165_∶VEGF_121_ is taken to be 92%∶8% [13].

We assume conservation of the density of receptors (ligated and unligated). In other words, at any time step, the density of receptors newly expressed on each membrane surface (luminal or abluminal) of the endothelial cells equals the density of receptors being internalized on that same surface. This assumption can be relaxed when more information on VEGF receptor dynamics become available.

Inter-compartment transport includes VEGF extravasation and intravasation (bidirectional transcapillary exchange) as well as lymphatic drainage of VEGF from the tissue to the blood. Unless specified otherwise, the vascular permeability to VEGF molecule is taken to be 4×10^−8^ cm/s in accordance with our previous model [9]. The lymphatic drainage in skeletal muscle of a healthy subject in the asleep, supine position has been reported to be between 1.7 and 2.5 µL/h/g [7],[14]. The total lymph flow rate at rest is estimated at 120 mL/hour [15], i.e., 2 cm^3^/min or 2.88 L/day (Table 5). As a first approximation, we assume that the removal rate of VEGF from our tissue compartment through the lymphatics corresponds to this lymph flow rate.

In our previous model, we assumed a VEGF clearance rate from plasma of 0.0206 min^−1^, corresponding to a VEGF half-life of approximately 34 min [9]. This was based on simple non-compartmental pharmacokinetic analysis of raw experimental data by Eppler et al. [16], which lumps together all routes of VEGF elimination from plasma. In order to better estimate the direct VEGF clearance rate from plasma (through protein degradation or kidney filtration, etc.), as distinct from alternate routes such as receptor-mediated metabolism or disappearance of extravasated VEGF by ligated receptors after biodistribution to muscle tissue compartments (which already has separate explicit mathematical representation in our model), we have adopted a theoretical clearance rate derived by Eppler et al. [16] through physiological mechanism-based compartmental biodistribution modeling, which predicts an elimination rate of 3.89 hr^−1^, corresponding to a clearance rate of VEGF from the plasma of 0.0648 min^−1^ (Table 5).

The VEGF plasma concentration in healthy subjects has been typically measured between 0.5–1.5 pM [17]. Unless specified otherwise, we maintain the average VEGF plasma concentration ∼1 pM in this model as a baseline. Note that the results are dependent on the expression of the receptors the quantitative knowledge of which in vivo is very limited.

## Results

### 

#### Free VEGF concentration in plasma is significantly altered by the change of VEGF clearance rate from plasma but not by the introduction of the lymphatic drainage

**Figure 2:**
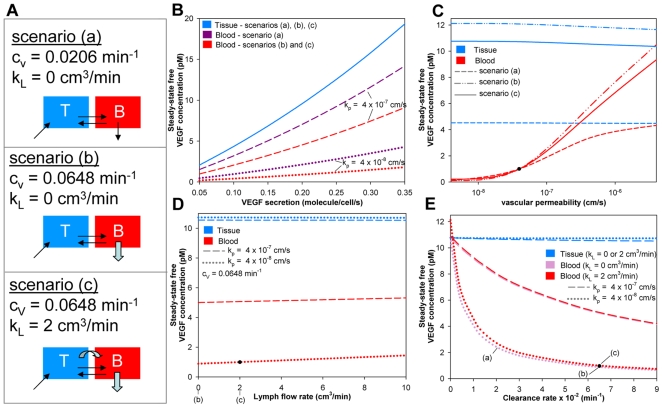
Effects of VEGF secretion, vascular permeability, lymph flow rate and clearance. A. Three scenarios are studied: scenario (a) our previous configuration from [9] (clearance rate *c_v_* = 0.0206 min^−1^); scenario (b) new clearance *c_v_* = 0.0648 min^−1^ in the absence of lymphatic drainage; scenario (c) introduction of the lymphatic drainage of VEGF *k_L_* = 2 cm^3^/min (120 mL/hour [15]) with the new clearance *c_v_* = 0.0648 min^−1^. B. Effect of VEGF secretion. Free VEGF concentration in the tissue is illustrated in blue. The purple curve corresponds to scenario (a) and the red curves to scenarios (b) and (c) for the plasma VEGF concentration. We consider two permeability rates *k_p_* = 4×10^−8^ cm/s (dotted curve) and 4×10^−7^ cm/s (dashed curve). C. Effect of vascular permeability to VEGF. Scenarios (a), (b) and (c) are represented as the dashed, dashed-dotted-dotted and solid curves respectively. The blue curve corresponds to the tissue while the red curve corresponds to the blood. D. Effect of lymph flow rate. We only consider scenarios (b) and (c) to look at the effect of adding the lymphatics to our model. We consider two permeability rates *k_p_* = 4×10^−8^ cm/s (dotted curve) and 4×10^−7^ cm/s (dashed curve). The blue curve corresponds to the tissue while the red curve corresponds to the blood. E. Effect of clearance rate. We consider two permeability rates *k_p_* = 4×10^−8^ cm/s (dotted curve) and 4×10^−7^ cm/s (dashed curve). The blue curve corresponds to the tissue. The pink curve corresponds to scenarios (a) and (b) and the red curve corresponds to the scenario (c) of Figure 2A for the plasma VEGF concentration.

We first evaluated the effects of changing the clearance rate from our previous study [9] and adding lymphatic transport for VEGF from the tissue to the vasculature in the absence of luminal receptors. The clearance rate for VEGF in the plasma was changed from 0.0206 min^−1^ (such that the VEGF half-life would be around 34 min in plasma) to 0.0648 min^−1^ (corresponding to a VEGF half-life of about 11 min). The flow rate of VEGF removal through lymphatics was taken to be 2 cm^3^/min. Figure 2A illustrates the three scenarios we considered: scenario (a) corresponding to our previous model [9] (clearance rate *c_V_* = 0.0206 min^−1^; no lymphatic drainage); scenario (b) is an intermediate step where the clearance rate of VEGF has changed in the absence of lymphatic drainage (clearance rate *c_V_* = 0.0648 min^−1^; no lymphatic drainage); scenario (c) corresponds to our new baseline (clearance rate *c_V_* = 0.0648 min^−1^; lymph flow rate *k_L_* = 2 cm^3^/min).

The secretion rate of VEGF in the tissue was varied from 0.05 to 0.35 molecule/cell/s (Figure 2B). VEGF was expressed at a ratio of 92%∶8% for VEGF_165_∶VEGF_121_ [13]. We considered two vascular permeabilities for VEGF (4×10^−8^ cm/s and 4×10^−7^ cm/s). For clarity, we do not show curves that overlap. The free VEGF concentration in the available interstitial fluid (tissue compartment) was not significantly altered by the change of clearance rate, the introduction of lymphatic drainage or the change of permeability (blue curve corresponding to scenarios (a), (b) and (c)). Increasing the clearance rate in the absence of lymphatic drainage lowered the free VEGF level in the plasma (purple and red curves comparing scenarios (a) and (b)). The introduction of the lymphatic removal of VEGF did not change the concentration of free VEGF significantly (red curve; scenarios (b) and (c)). This result is in contrast to our previous study [7], where we examined higher and a larger range of lymphatic drainage rates for soluble proteins as a function of muscle activity (lymphatic pump), which significantly affected free VEGF concentration gradients between plasma and tissue interstitium. As mentioned in our previous study [9], increasing the vascular permeability to VEGF induced an increase of the plasma free VEGF concentration (dotted vs. dashed curves) without significantly altering the interstitial free VEGF level.

#### Increasing the vascular permeability to VEGF significantly affected the free VEGF concentration in blood but not in tissue

The different curves represented in Figure 2C illustrate the VEGF concentration responses in available interstitial fluid and plasma to vascular permeability for the three scenarios illustrated in Figure 2A. Similar to that used in our previous study [9], the baseline of each simulation was taken such that, at a vascular permeability of 4×10^−8^ cm/s, about 1 pM of free VEGF was present in the plasma (black dot). This means that the secretion rate had to be tuned for each simulation. The total VEGF secretion rates were 0.1126 (dashed curve), 0.2634 (dashed-dotted-dotted curve) and 0.2390 molecule/cell/s (solid curve) for scenarios (a), (b) and (c) respectively. We then varied the vascular permeability to VEGF from 4×10^−9^ to 4×10^−6^ cm/s. In all scenarios, free VEGF concentration in the available interstitial fluid was found to remain fairly constant over the range we considered. Increasing the clearance rate required a higher free VEGF concentration in the available interstitial fluid (blue dashed vs. dashed-dotted-dotted curves, i.e., scenario (a) vs. (b)). This is because a higher secretion was required to reach 1 pM of free VEGF at steady state in plasma when the clearance rate was increased. The introduction of the VEGF removal through the lymphatics reduced the free VEGF level in the tissue (dashed-dotted-dotted vs. solid curves, i.e., scenario (b) vs. (c)) since VEGF was drained from the available interstitial fluid into the plasma thus requiring a lower secretion rate to attain the 1 pM in the plasma. A similar behavior was noted in the blood for a range of vascular permeability higher than 4×10^−8^ cm/s.

In our previous study [9], three regions were identified: for a vascular permeability higher than 4×10^−6^ cm/s, the free VEGF concentration in the plasma converged to that in the available interstitial fluid; for a vascular permeability lower than 4×10^−8^ cm/s, free VEGF concentration in the plasma was fairly constant and close to zero; and for a vascular permeability range between 4×10^−8^ to 10^−6^ cm/s, the free VEGF concentration was approximately proportional to vascular permeability. These last two regions could still be observed in Fig. 2C for scenarios (b) and (c). However, it was clear that the new clearance rate and the introduction of VEGF lymphatic drainage required a higher vascular permeability for the free VEGF concentration in plasma to equal that in the tissue (as compared to scenario (a)). This is due to the fact that a higher net transport of VEGF from the tissue into the blood compartment is required to compensate the loss of VEGF with a higher clearance rate.

#### Free VEGF concentration in the plasma is affected by the vascular permeability as compared to the lymphatic drainage

Figure 2D shows the variation of free VEGF concentration in the tissue and plasma with the lymph flow rate. The removal rate of VEGF through lymphatics was varied from 0 to 10 cm^3^/min, i.e., 0 to 600 mL/h or 0 to 14.4 L/day. The baseline was taken so that, at a vascular permeability of 4×10^−8^ cm/s, a clearance rate of 0.0648 min^−1^ and a lymph flow rate of 2 cm^3^/min, about 1 pM of free VEGF was present in the plasma (black dot on Figure 2D). Free VEGF level does not change significantly in the tissue compartment. Within the tested range, increasing the rate at which VEGF is removed through the lymphatics increased the free VEGF concentration in the plasma. However, this augmentation was less noticeable when the vascular permeability was increased 10-fold (dotted vs. dashed curves): for a vascular permeability of 4×10^−8^ cm/s, the free VEGF concentration in plasma varied from 0.89 to 1.44 pM (a 63% increase), whereas at a vascular permeability of 4×10^−7^ cm/s, it varied from 5.01 to 5.31 pM (a 6% increase). This is due to competition between the transendothelial exchange of free VEGF (net permeability) and the lymphatic drainage of VEGF.

#### In the absence of clearance, the lymphatic drainage of VEGF can invert the gradient of free VEGF concentration across the endothelial barrier

We varied the clearance rate for VEGF from 0 (which corresponds to an infinite half-life of VEGF) to 0.09 min^−1^ (about 8-minute VEGF half-life). The results are shown in Figure 2E. The free VEGF concentration in the available interstitial fluid was fairly insensitive to the change of clearance. However, the free VEGF level in plasma was significantly affected by the variation of clearance. The introduction of the lymphatics (red vs. light pink curves) did not significantly change the free VEGF concentration for most of the range of clearance rate studied. However, when the lymphatic drainage was introduced and the clearance of VEGF was set to zero (infinite half-life for VEGF in the plasma), the concentration of free VEGF was higher in the plasma than in the available interstitial fluid regardless of the vascular permeability to VEGF for the range we checked. In such case, the gradient across the endothelial cells (i.e., between the tissue and the blood compartments) was inverted. In the absence of the lymphatics and when the clearance was set to zero, the VEGF concentrations were equal, as expected. This effect was not as drastic for a vascular permeability of 4×10^−7^ cm/s due to increased equilibration of the compartments by the intravasation/extravasation of VEGF.

#### Effects of VEGF receptors on free VEGF concentrations in available interstitial fluid and in plasma

**Figure 3:**
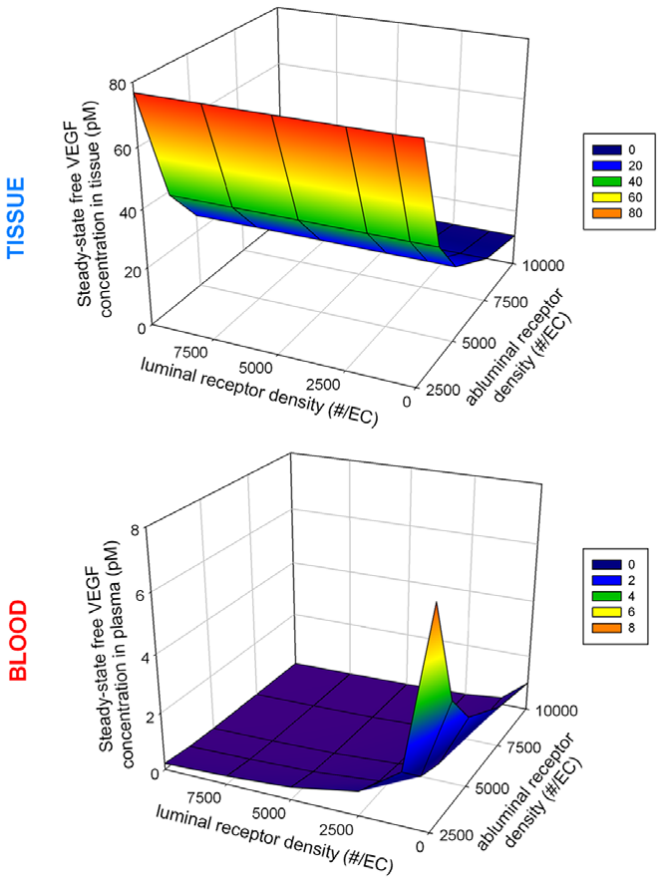
Free VEGF concentrations in tissue and blood as a function of the receptor density. The secretion rate is fixed so that, at a vascular permeability of 4×10^−8^ cm/s, 1 pM of free VEGF is present in the plasma (no luminal receptors; 10,000 abluminal receptors of each species per endothelial cell). VEGF secretion rate is 0.2390 molecule/cell/s. The density of abluminal and luminal receptors was varied from 0 to 10,000 receptors per endothelial cell surface. Note that no steady state could be reached at such secretion rate in the absence of abluminal receptors. The free VEGF concentration in the available interstitial fluid was constant over the range of luminal receptor density and decreased exponentially with the density of abluminal receptors. The free VEGF concentration in the plasma was significantly changed when the density of receptors was low.

We varied the density of receptors on the luminal and abluminal surfaces on the endothelial cells lining the capillaries and looked at the change of free VEGF concentrations in the tissue and in the blood compartments, as shown in Figure 3. The baseline was taken to be 1 pM of free VEGF concentration in the plasma in the absence of luminal receptors and in the presence of 10,000 VEGFR1, 10,000 VEGFR2 and 10,000 NRP1 on the abluminal side of the endothelial cells [9]. Note that using a single-compartment model of skeletal muscle we previously conducted a detailed sensitivity analysis on the effect of receptor density on VEGF distribution [18]. The vascular permeability was fixed at 4×10^−8^ cm/s. The clearance rate was 0.0648 min^−1^ and the lymph flow rate was set at 2 cm^3^/min. In this set of experiments, the secretion rate was not changed across the simulations and the total VEGF secretion rate was 0.2390 molecule/cell/s (with a VEGF expression rate ratio VEGF_121_: VEGF_165_ of 92%∶8%, i.e., VEGF_165_ secretion rate = 0.2199 molecule/cell/s and VEGF_121_ secretion rate = 0.0191 molecule/cell/s). Unless specified otherwise, the density of receptors denotes the density of each species of receptors. For example, “5,000 abluminal receptors per endothelial cell” means “5,000 of each species (VEGFR1, VEGFR2, and NRP-1) per endothelial cell located on the abluminal surface.” The luminal receptors were varied from 0 to 10,000 receptors per endothelial cell. However, the fixed secretion rate was too high to reach a steady state for an abluminal receptor density smaller than 2,500 receptors per endothelial cell. Increasing the density of luminal receptors did not affect the free VEGF concentration in the available interstitial fluid but drastically decreased that in the plasma. Increasing the density of abluminal receptors decreased the concentration of total free VEGF in both the tissue and the blood compartments. This is due to receptor binding: the higher the receptor density, the smaller the free VEGF concentration.

#### When the plasma concentration of free VEGF is fixed, free VEGF concentration in the available interstitial fluid is directly proportional to the density of luminal receptors

**Figure 4:**
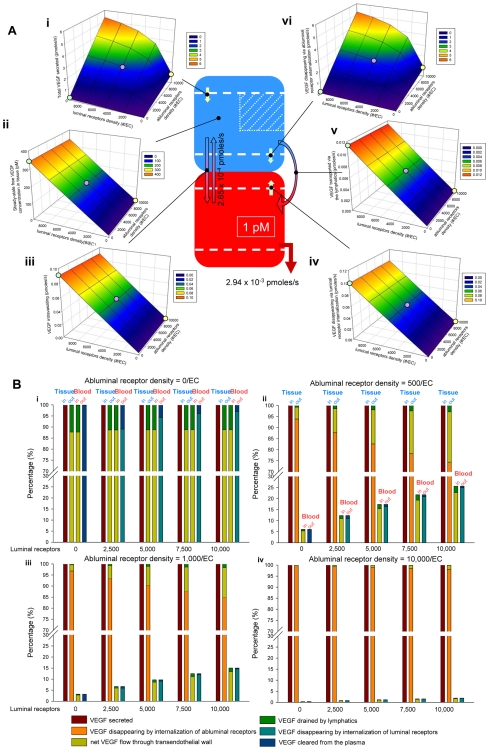
Flow diagrams for a fixed concentration of free VEGF in the plasma. A. The inflows and outflows are expressed in pmoles/s. The density of luminal and abluminal receptors was varied from 0 to 10,000 per endothelial cell. Free VEGF concentration in the plasma was fixed at 1 pM for a vascular permeability of 4×10^−8^ cm/s. From top left, counter-clockwise: i. VEGF secreted per parenchymal cell; ii. free VEGF concentration in the tissue (in pM); iii. VEGF intravasating; iv. VEGF disappearing through internalization of the luminal receptors to which it binds; v. VEGF drained through the lymphatics; vi. VEGF disappearing through internalization of abluminal receptors to which it binds. VEGF extravasating and VEGF cleared from the plasma are constant over the course of the simulations due to the fixed free VEGF concentration in the plasma and equal to 2.65×10^−4^ and 2.94×10^−3^ pmoles/s respectively. The yellow dot corresponds to the configuration of 10,000 abluminal receptors and no luminal receptors. The purple dot identifies an equal density of receptors on luminal and abluminal surfaces of the endothelial cells (5,000 receptors on each side per endothelial cell). The green dot corresponds to the case of 10,000 luminal receptors and no abluminal receptors. B. Flows normalized to VEGF secretion for different luminal receptor densities: i. no abluminal receptors; ii. 500 abluminal receptors per EC; iii. 1,000 abluminal receptors per EC; iv. 10,000 abluminal receptors per EC. EC = endothelial cell.

The plasma free VEGF concentration was fixed at 1.00 pM at a vascular permeability of 4×10^−8^ cm/s, a plasma clearance rate of 0.0648 min^−1^ and a lymph flow rate of 2 cm^3^/min. Figure 4A summarizes the dependence of the flows of VEGF (Figures 4Ai, iii, iv, v, vi, in pmoles/s) and the free VEGF concentration in the available interstitial fluid (Figure 4Aii) on the receptor densities on the luminal and abluminal surfaces of the endothelial cells. Note that, for each simulation, we therefore readjusted the VEGF secretion rate.

First, keeping the free VEGF concentration in plasma constant fixes some of the outflows from the blood compartment since they are directly proportional to the VEGF concentration. In other words, the VEGF cleared from the blood was then constant throughout the simulations (2.94×10^−3^ pmoles/s) and so was the rate of VEGF extravasation (2.65×10^−4^ pmoles/s) as indicated on the model diagram in Figure 4A. VEGF disappearing by internalization of luminal ligated receptors (blood compartment) was proportional to the density of receptors on the luminal surface of the endothelial cells (Figure 4Aiv). This was explained by the fact that the internalization terms of ligated receptors in the equations were expressed as 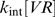. Since we assumed a fixed total density of receptors at any time-step, i.e., 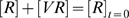, VEGF disappearing by internalization of ligated receptors was linearly dependent on the density of luminal receptors. VEGF flow from the tissue to the blood compartment (intravasation and lymphatic removal of VEGF) was also found to be directly proportional to the density of luminal receptors (Figures 4Aiii and 4Av respectively). Although this may be surprising, it follows from the balance of the inflows and outflows in the blood compartment.
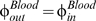


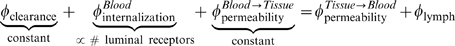
so 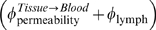 is also proportional to the density of receptors on the luminal endothelial surface. Since these two outflows differ only by a constant of proportionality, each term is therefore linearly dependent on the density of luminal receptors. These outflows are also directly proportional to the concentration of free VEGF in the available interstitial fluid, explaining the linear dependence of free VEGF concentration in the tissue compartment on the density of receptors on the luminal endothelial surface (Figure 4Aii). Finally, VEGF secreted (Figure 4Ai) and VEGF disappearing by internalization of its bound receptors on the abluminal surface of the endothelial cells (Figure 4Avi) reach saturation when the receptor density on the luminal endothelial surface is high enough to push the free VEGF concentration in the available interstitial fluid higher than K_d_ of VEGF and its receptors (i.e., the saturation occurs when free VEGF in the available interstitial fluid is several times higher than K_d_(VEGF,VEGFR)). Interestingly, VEGF secretion and internalization through abluminal receptors were, however, linearly dependent on the density of receptors on the abluminal endothelial surface. This is mainly because the free plasma VEGF concentration was constant over the tested range of abluminal receptors.

Figure 4B shows the VEGF flows normalized to VEGF secretion. The ratios are given in percentages. In the absence of abluminal receptors (Figure 4Bi), most of the free VEGF intravasates (>85%) regardless of the luminal receptor density while, in the presence of abluminal receptors, most VEGF disappears by internalization of abluminal ligated receptors (Figures 4Bii–iv). When abluminal receptors are present, less than 25% of VEGF that has been secreted effectively enters the blood compartment. Increasing the luminal receptor density yields more VEGF entering the blood by intravasation. This is mainly due to the fact that the model requires a higher secretion rate to balance the increase in receptor density and internalization. Finally, unless there are no luminal receptors (in which case free VEGF disappears from the plasma by clearance), most free VEGF leaves the blood by internalization of the luminal ligated receptors.

#### VEGF disappearing by internalization of abluminal ligated receptors is proportional to VEGF secretion

**Figure 5:**
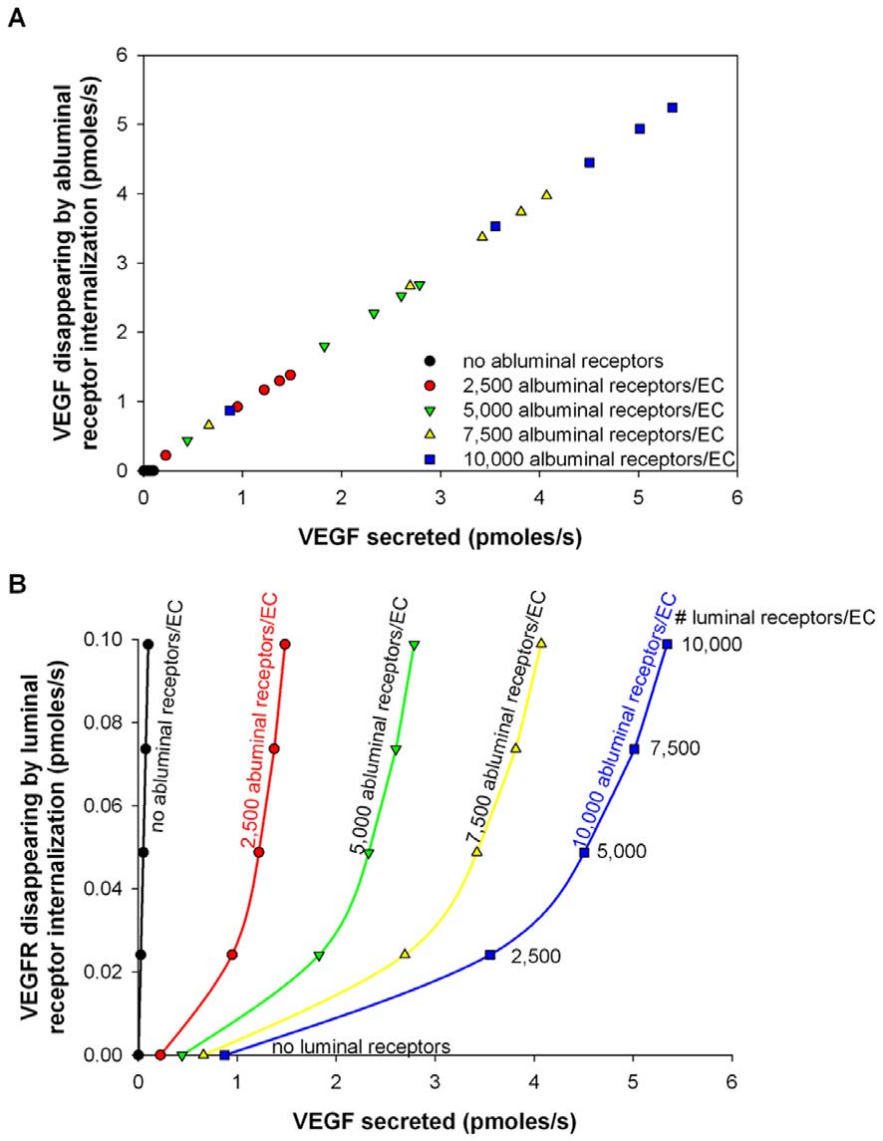
Flows of VEGF disappearing upon ligated receptor internalization as a function of the VEGF secreted. The setup is similar to that in Figure 4. A. Linear relationship between the VEGF secreted and the VEGF disappearing via internalization of VEGF-bound abluminal receptors. B. Non-linear relationship between the VEGF secreted and the VEGF disappearing via internalization of the luminal receptors it has bound to. Black circles: no abluminal receptors; red circles: 2,500 abluminal receptors/endothelial cell; green triangles: 5,000 abluminal receptors/endothelial cell; yellow triangles: 7,500 abluminal receptors/endothelial cell; blue squares: 10,000 abluminal receptors/endothelial cell.

Noting similarity between Figures 4Ai and 4Avi, we mathematically showed that VEGF secretion and VEGF disappearing by internationalization of the abluminal receptors are proportional to each other as illustrated in Figure 5A. The following mathematical relationship was derived:
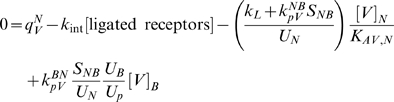
(5)


This equation holds true not only for total VEGF but also for each VEGF isoform individually and corresponds to the conservation of VEGF molecules in the tissue compartment.

No linear relationship was found when looking at VEGF disappearing by internalization of luminal ligated receptors (blood compartment) in relation to the VEGF secreted as shown in Figure 5B. However, the density of luminal receptors fixed the internalization of ligated luminal receptors (dotted lines) but the density of abluminal receptors dictated the form of the relationship with secreted VEGF (solid lines).

#### When the receptors are evenly distributed on the endothelial cell surface, the VEGF plasma clearance and extravasation are minimized

**Figure 6:**
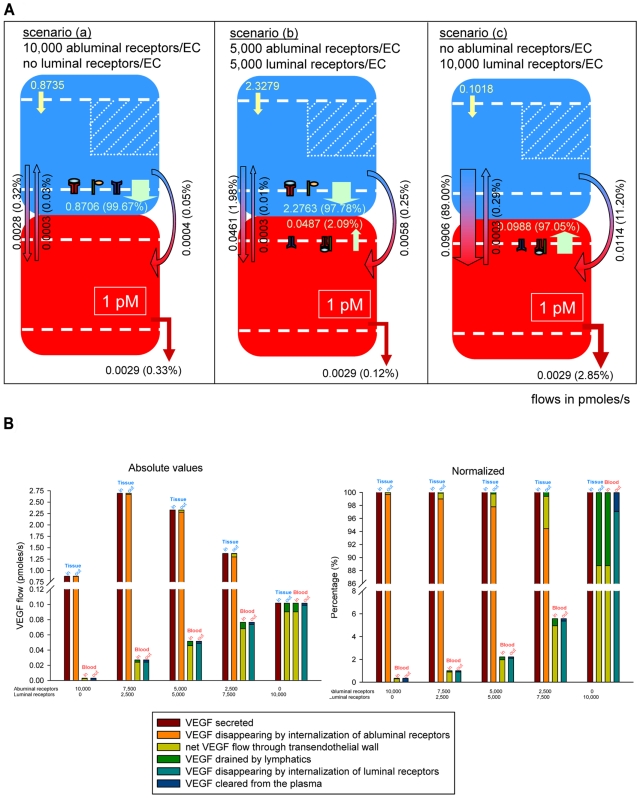
Flow diagrams for a total receptor density of 10,000 per endothelial cells. A. The density of total (luminal and abluminal) receptors per endothelial cell is fixed at 10,000 (VEGFR1∶VEGFR2∶NRP1 expression is 1∶1∶1). Scenario (a): all the receptors are located on the endothelial abluminal surface (yellow circle on Figure 4A); scenario (b): the receptors are evenly distributed between the luminal and abluminal endothelial surface (purple circle on Figure 4A); scenario (c): all the receptors are located on the endothelial luminal surface. Numbers represent absolute values of VEGF flows expressed in pmoles/s. Percentages of VEGF secretion in parentheses. B. Generalization of particular cases shown in A. The density of receptors varies between 0 and 10,000 receptors per endothelial cell surface. The total receptor density is fixed at 10,000 receptors per endothelial cell. Left: absolute values; Right: percentages of VEGF secretion.

We next examined how the ratio of receptor densities on the luminal vs. abluminal endothelial surface can impact transport. We fixed the density of total receptors (luminal and abluminal) to 10,000 per endothelial cell. Figure 6A illustrates three configurations. Scenario (a) represents the case where all the receptors are located on the abluminal side (tissue compartment), i.e., 10,000 receptors on the abluminal endothelial surface and no luminal receptors in this representation. This corresponds to the yellow dots on Figure 4A. Scenario (b) represents the case where the receptors are evenly distributed between the abluminal and luminal surfaces of the endothelial cells, i.e., 5,000 receptors of each species are present in each compartment. This corresponds to the purple dots on Figure 4A. Finally, scenario (c) illustrates the case where all the receptors are located on the luminal endothelial surface, i.e., 10,000 receptors on the luminal surface of the endothelial cells and no abluminal receptors. This corresponds to the green dots on Figure 4A. We investigated how the inter- and intra-compartment flows of VEGF vary between the configurations. We found that the transport of VEGF by intravasation, lymphatic drainage, and internalization of luminal ligated receptors increase when the receptors are “redistributed” from the abluminal to the luminal surface of the endothelial cells. However, to maintain 1 pM of free VEGF in the plasma, VEGF secretion varied significantly between the three scenarios considered. For comparison purposes, we therefore normalized the flows to VEGF secretion. These normalized inflows and outflows are noted in terms of percentages of VEGF secretion as indicated in parentheses in Figure 6A. Although the clearance and the extravasation of VEGF were constant in terms of absolute values (at 0.0029 and 0.0003 pmoles/s respectively, as shown in Figures 4A and 6A), the corresponding normalized values became minimal when the receptors were evenly distributed between the luminal and the abluminal surfaces of the endothelial cells (scenario (b) in Figure 6A).

Figure 6B generalizes these findings for more possible configurations of a total of 10,000 receptors (of each species) expressed per endothelial cell. The ratios of receptors on abluminal∶luminal endothelial surfaces are 10,000∶0 (scenario (a) in Figure 6A); 7,500∶2,500; 5,000∶5000 (scenario (b)); 2,500∶7,500; and 0∶10,000 (scenario (c)). The net transendothelial VEGF flow is the difference of VEGF intravasating and VEGF extravasating. As long as abluminal receptors are present, most secreted VEGF disappears by internalization upon binding to the abluminal receptors. In the absence of abluminal receptors, intravasation is the main route by which VEGF leaves the interstitial fluid. The fraction of VEGF entering the plasma is, in all cases, mainly driven by the permeability rather than by lymphatics.

#### VEGF is sequestered in the extracellular matrix when the receptors are evenly distributed between the abluminal and the luminal surfaces of the endothelial cells

**Figure 7:**
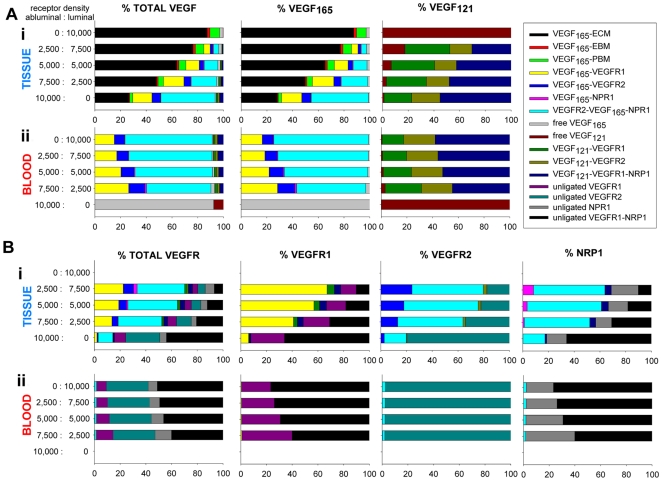
VEGF distribution and VEGFR occupancy for a total receptor density of 10,000 per endothelial cells. The scenarios correspond to those in Figure 6B. A. VEGF distribution. i. tissue; ii. blood. Columns from left to right: total VEGF distribution, VEGF_165_ distribution, VEGF_121_ distribution. In the absence of luminal receptors (bottom rows in i. and ii.), most VEGF bridges VEGFR2-NRP1 in the tissue and VEGF_165_∶VEGF_121_ in the plasma is 92%∶8% similar to that of the isoforms expressions in the tissue. When abluminal receptor density decreases, VEGF is increasingly sequestered in the extracellular matrix (ECM, PBM, EBM) in the tissue compartment and VEGF_165_ bridges VEGFR2-NRP1 in the plasma. B. VEGFR occupancy. i. tissue; ii. blood. Columns from left to right: total VEGFR occupancy, VEGFR1 occupancy, VEGFR2 occupancy, NRP1 occupancy. When the receptors are distributed on one side of the endothelial cells (either luminal or abluminal), most of the VEGF receptors are in the form of VEGFR1-NRP1 complex while most of VEGFR2 is in its free state (bottom row in i. and top row in ii.). When the receptors are distributed evenly between the endothelial cells luminal and abluminal surfaces, the luminal receptor occupancy remains unchanged (top and middle rows in ii.) but the VEGF receptor occupancy in the tissue shifts towards VEGF_165_ bridging VEGFR2-NRP1 (bottom and middle rows in i.).

Figure 7A shows the distributions of VEGF when the total density of total receptors (abluminal + luminal) was fixed at 10,000 receptors per endothelial cell. The ratios of receptors on abluminal∶luminal endothelial surfaces are 10,000∶0 (corresponding to scenario (a) in Figure 6A); 7,500∶2,500; 5,000∶5000 (scenario (b)); 2,500∶7,500; and 0∶10,000 (scenario (c)). In the absence of luminal receptors (scenario (a) – bottom rows in Figures 7Ai and 7Aii), most VEGF is in the form of the triplet VEGFR2-VEGF_165_-NRP1 (42%) while about a quarter of VEGF is sequestered in the interstitium. In the blood, free VEGF_165_ accounts for 92% of the total population of VEGF. When there is an equal density of receptors on the abluminal and luminal sides (“even distribution” – scenario (b); middle rows), about 75% of VEGF in the tissue is sequestered in the interstitium. Interestingly, in the blood, most of the VEGF_165_ is bound to VEGFR2 (or bridges VEGFR2-NRP1) while most of VEGF_121_ is bound to VEGFR1 (or the VEGFR1-NRP1 complex). These results were even more pronounced when all the receptors were located on the luminal side (scenario (c) – top rows). One striking result was that free VEGF_121_ represented between 0.07% to 0.25% of the total VEGF distribution in the tissue (Figure 7Ai), and dropped from 8% to 0.06% of the total VEGF distribution in the blood (Figure 7Aii) when the population of receptors “shifted” from the abluminal to the luminal surface of the endothelial cell. Together with the results from Figure 6, this means that most of the VEGF_121_ secreted in the tissue was cleared from the plasma or disappeared by the internalization of the luminal receptors while blood VEGF_165_ is in a form of the triplet VEGFR2-VEGF_165_-NRP1. The increase of free VEGF_121_ in the tissue can also be explained by the increasing sequestration of VEGF_165_ by the ECM. Figure 7A demonstrates that the location of receptors on the endothelial cells can drastically affect the VEGF distribution in the plasma and in the tissue.

Figure 7B shows the receptor occupancies. In the absence of receptors on one surface of the endothelial cells (scenario (a) or (c) – top and bottom rows in Figures 7Bi and 7Bii), most of the remaining receptors is in the form of the VEFGR1-NRP1 complex while VEGFR2 is in its free state. This result does not change significantly on the luminal endothelial surface when an equal density is present on both luminal and abluminal surfaces of the endothelial cells (scenario (b) – middle rows). However, in the tissue, the occupancy of the receptors is “shifted” towards the triplet VEGFR2-VEGF_165_-NRP1 which causes the population of unbound VEGFR2 to be significantly reduced. In such case, VEGFR1 is mainly bound by VEGF_165_ in the tissue.

#### In the tissue, more VEGF is bound to the ECM and less is bound to the abluminal receptors as the density of abluminal receptors decreases and the density of luminal receptors increases

**Figure 8:**
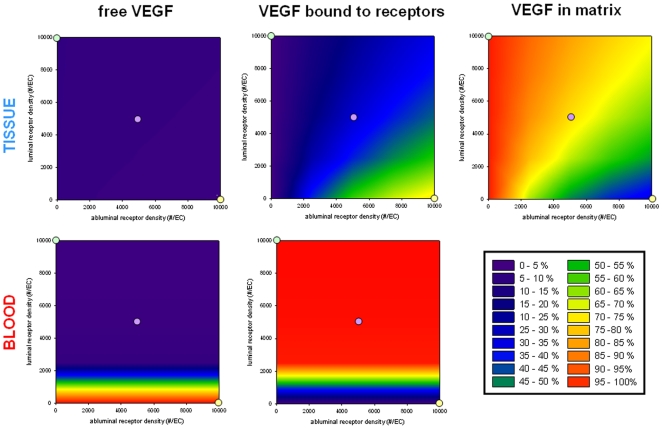
VEGF distributions in the tissue and in the blood. The setup is similar to that in Figure 4. Top row: VEGF distribution in the tissue; bottom row: VEGF distribution in the blood. The first column corresponds to the percentage of free VEGF; the second column the percentage of VEGF bound to the receptors; the third column the percentage of VEGF bound to the extracellular matrix and basement membranes.

In the plasma, the amount of VEGF bound to the luminal receptors is insensitive to the density of abluminal receptors when VEGF plasma concentration is fixed. Figure 8 illustrates the distribution of VEGF (free, bound to the receptors, and sequestered in the matrix) when the receptor density varies from 0 to 10,000 receptors per endothelial cell on each surface of the endothelial cell. The three yellow (scenario (a)), purple (scenario (b)) and green (scenario (c)) dots correspond to the cases studied in Figure 6A. We found that only a small fraction of VEGF is free in the available interstitial fluid. Most VEGF is either bound to the abluminal receptors or sequestered in the matrix. VEGF is more and more bound to the receptors and less and less sequestered in the matrix when the density of luminal receptors decreases and the density of abluminal receptors increases. In the blood, VEGF becomes more bound to the receptors with the increasing density of luminal receptors but the general VEGF distribution in this compartment does not significantly vary with the abluminal receptor density across the interval tested.

#### Because the VEGF secretion occurs in the tissue, VEGF binding to VEGFR1 and VEGFR2 takes place predominantly on the abluminal surface of the endothelial cells, regardless of the ratio of the receptors on each surface of endothelial cell

**Figure 9:**
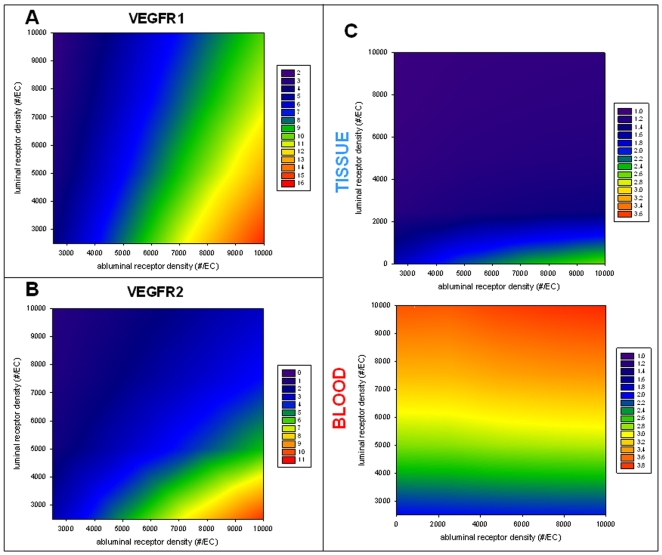
VEGF signaling. A. Ratio [VEGF bound to abluminal VEGFR1]/[VEGF bound to luminal VEGFR1]. B. Ratio [VEGF bound to abluminal VEGFR2]/[VEGF bound to luminal VEGFR2]. C. Ratio [VEGF bound to VEGFR2]/[VEGF bound to VEGFR1]. Top row: tissue; bottom row: blood.

We next looked at how much VEGF is bound to the receptors on the abluminal surface of the endothelial cells as compared to how much VEGF is bound to receptors on the luminal surface of the endothelial cells. The ratio [VEGF bound to abluminal VEGFR1]/[VEGF bound to luminal VEGFR1] is shown in Figure 9A and the ratio [VEGF bound to abluminal VEGFR2]/[VEGF bound to luminal VEGFR2] is illustrated in Figure 9B. For most cases, these two ratios were higher than 1, meaning that the amount of VEGF bound to VEGFR1 or VEGFR2 was higher on the abluminal than on the luminal surface of the endothelial cells. This is explained by the fact that the VEGF secretion occurs in the tissue, leading to a VEGF gradient from the tissue to the blood compartment. However, a small region revealed more binding on the luminal side (ratio <1) for VEGFR2 (Figure 9B). This small region corresponds to low abluminal and high luminal receptor densities. This region reveals receptor binding for VEGFR1 higher on the abluminal endothelial surface (Figure 9A). Therefore, at low abluminal and high luminal receptor densities, there is more binding to VEGFR1 on the abluminal surface and more binding to VEGFR2 on the luminal surface. This particular region calls for experimental exploration.

#### VEGF signaling

We next compared how much VEGF is bound to VEGFR1 as compared to bound to VEGFR2. VEGFR2 is pro-angiogenic, whereas VEGFR1 is anti-angiogenic or modulatory [19], thus the ratio might represent pro- vs. anti-angiogenic signaling. Figure 9C illustrates the ratio [VEGF bound to VEGFR2]/[VEGF bound to VEGFR1] in each compartment (i.e., the abluminal and luminal surfaces of the endothelial cells). If this ratio is higher than 1, then VEGF is predominantly bound to VEGFR2. Conversely, if the ratio is lower than 1, then VEGF is predominantly bound to VEGFR1. Figure 9C shows that the two ratios are always higher than 1 in both the tissue and the blood compartments for our region of interest, meaning that the VEGF binds more to VEGFR2 than to VEGFR1, even though the total VEGFR1 and VEGFR2 densities are assumed equal (as mentioned previously, VEGFR1∶VEGFR2∶NRP1 are expressed on each endothelial cell surface in 1∶1∶1 ratio). Since the K_d_ for VEGF-VEGFR2 is three times higher than the K_d_ for VEGF-VEGFR1 binding, this means that this cannot be the consequence of a higher binding affinity for VEGFR2 but rather of the neuropilin-1 presence. We also note that the higher the receptor density on the abluminal endothelial surface, the more binding to VEGFR2 (as compared to VEGFR1) on this same surface. However, the magnitude of the ratio is much higher on the luminal endothelial surface (blood compartment) than on the abluminal endothelial surface (tissue compartment). This is most likely because only a small fraction of free VEGF intravasates (Figure 8).

## Discussion

This extension of our previous model [9] is useful for exploring the effects of luminal vs. abluminal distribution of VEGF receptors on the endothelial surfaces. We have shown that such configurations can drastically affect the VEGF profile in the tissue and in the blood.

**Figure 10:**
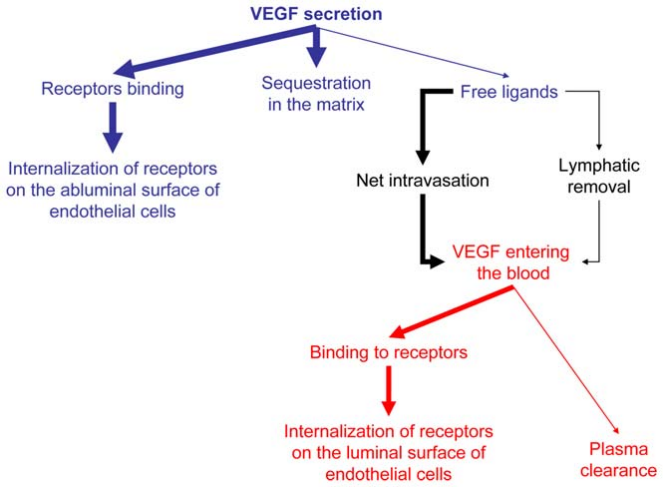
Summary of VEGF transport in the body. VEGF is secreted by parenchymal cells in the tissue. Most of VEGF is sequestered in the extracellular matrix or binds to the abluminal receptors and disappears by VEGF-bound receptor internalization. A small fraction (free VEGF) is transported from the available interstitial fluid to the plasma (mostly through the permeability route rather than by the lymphatics). Upon entering the blood, free VEGF either binds to luminal receptors and disappears by VEGF-bound receptor internalization or is cleared from the plasma.

First, we have shown that the removal of clearance in the presence of lymphatics could reverse the free VEGF gradient between the tissue and the blood compartments. Such a situation might correspond to certain pathological conditions, but the simulation is also instructive as a characterization of the VEGF transport system. However, it is important to note that our current model does not explicitly include the convective component of transvascular permeability and such addition could attenuate the predicted gradient reversal. Secondly, at a fixed VEGF secretion rate, the free VEGF in the available interstitial fluid is much higher than that in the plasma. When the free VEGF concentration in the plasma is constant (∼1 pM), VEGF extravasation and plasma VEGF clearance over time are constant over the range of receptors we studied. We have found that the amount of VEGF disappearing by internalization of luminal receptors to which it binds, the amount of VEGF extravasating and the amount of VEGF removal from lymphatic drainage are all proportional to the luminal receptor density but insensitive to the abluminal receptor density. We have established a mathematical relationship between the amount of VEGF secreted and VEGF disappearing by internalization of abluminal receptors. Thirdly, we can summarize the VEGF transport between the tissue and the blood as shown in Figure 10. VEGF is secreted in the tissue. Depending on the receptor density on the abluminal and luminal endothelial surfaces, VEGF is mainly either sequestered by the matrix or binds to abluminal receptors. Upon binding, VEGF disappears by internalization of the abluminal receptors it has bound to. Only a small fraction (free ligands) enters the blood compartment (mainly by intravasation rather than lymphatic drainage). VEGF then disappears either by internalization of receptors located on the luminal endothelial surface to which they bind or, when the receptor densities are very low, by plasma clearance. This overall transport explains why, regardless of where the receptors are expressed on the endothelial cells (abluminal vs. luminal surfaces), the binding to the receptors occurs more in the tissue than in the plasma (since a higher concentration of free ligands is available in this compartment – due to secretion – as compared to the free VEGF in the blood). However, our simulations have revealed that for high abluminal and low luminal receptor densities, VEGF can bind “preferentially” to VEGFR1 on the abluminal surface and to VEGFR2 on the luminal surface of the endothelial cells. This result requires experimental exploration. In particular, this result shows that quantification of luminal vs. abluminal receptors can be crucial in understanding VEGF signaling in both physiological and pathological conditions. Finally, our simulations reveal that VEGF binds “preferentially” to VEGFR2 compared to VEGFR1. If VEGFR2 is shown to be pro-angiogenic and VEGFR1 is shown to be anti-angiogenic, then we can conclude that, overall, the signaling is mainly pro-angiogenic regardless of the receptor distribution on the endothelial cells.

Since VEGF receptor distribution between the abluminal and luminal endothelial surfaces plays such an important role, it would be interesting to investigate if some pathologies could be explained by decreased receptor expression or internalization. For example, in our previous model, we had shown that an increase in VEGF vascular permeability or secretion could not solely explain the increase of free VEGF concentration in plasma seen in cancer patients [9]. It could be interesting to see if deregulated receptor expression could explain the plasma VEGF increase in cancer (as compared to healthy subjects). The present model suggests, for example, that VEGF could intravasate in high proportion if the amount of VEGF disappearing by internalization of bound receptors decreases, i.e., if the internalization rate of the receptors or if the receptors expression decreases.

The present model also suggests that, since most of VEGF disappears via internalization of bound receptors (whether on the luminal or abluminal endothelial surface), the increase of internalization of receptors could potentially decrease VEGF signal transduction. This could be done either by increasing the internalization rate of the already-existing receptors or by bioengineering cells expressing VEGF receptors which would have the property of having a high binding affinity for VEGF as well as a higher internalization rates than endothelial cells. Decreasing the VEGF signal transduction of endothelial cells could have potential therapeutic applications.

For a complex system such as the VEGF receptor-ligand interactions and transport considered, it is necessary to add elements and further increase the degree of complexity step by step in order to understand the effect of each factor. We can outline further steps in refining the model. First, the model has looked at the effect of the receptors in the proportion 1∶1∶1 for VEGFR1∶VEGFR2∶NRP1. It would also be of interest to see how unequal ratios of receptors can influence the distribution and concentration of VEGF, especially when experimental data on receptor distribution in vivo become available. Secondly, at the moment, the model considers two isoforms of VEGF: VEGF_121_ and VEGF_165_. Other isoforms could be added to the computational model when new quantitative information becomes available. The model could also include neuropilin-2 which could compete for VEGF. Thirdly, the introduction of soluble VEGFR1 (sFlt-1) would also be of interest, especially since recent results have shown that sFlt-1 can serve as an additional means for VEGF to be transported from the plasma into the tissue [7]. In that study, we hypothesized that the anti-angiogenic potential of sVEGFR1 may stem from its dominant-negative heterodimerization with cell surface VEGFRs and predicted that the circulating (plasma) level of sVEGFR1 is significantly higher than its interstitial concentrations, which could imply that sVEGFR1 may have a greater modulatory influence on luminal VEGFRs than abluminal VEGFRs [7],[11].

Platelets have been shown to be significant reservoirs of VEGF in the blood circulation. It would be interesting to include such elements into the model. Again, quantification of luminal receptors would be crucial, especially since platelets have been shown to sequester large amounts of VEGF and release VEGF from α-granules [20],[21].

Similarly, the body tissue compartment was considered to have the properties of skeletal muscle. It could be important to distinguish between highly vascularized and relatively avascular organs, as well as elements with varying rates of lymphatic drainage. This would require experimental data on VEGF secretion and other tissue characteristics that at present are poorly known. Furthermore, luminal and abluminal receptors may not be equally accessible by VEGF possibly because of endothelial cell polarity: basement membrane on the abluminal side and glycocalyx on the luminal side.

A current assumption was the conservation of total (free and bound) density of receptors at each time step. In other words, we assumed that the internalization of receptors was equal to the receptor insertion per abluminal or luminal endothelial surface for each time point. Relaxing such assumptions and replacing them by the experimentally-based receptor dynamics would make the model more accurate.

In our model, we assumed that the vascular permeability was fixed. In reality, VEGF, also known as VPF (vascular permeability factor), plays an important role in regulating permeability [22]. An addition to the model would be to determine a quantitative relationship between the vascular permeability and the concentration of VEGF and include that relationship in the model.

Our study has shown that quantification of luminal vs. abluminal receptors could be very useful to better understand VEGF signaling and the mechanisms underlying VEGF-dependent diseases as well as angiogenesis and will motivate experimental exploration.

## Acknowledgments

The authors thank Elena Rosca, Amina Qutub, Emmanouil Karagiannis, Princess Imoukhuede, Jacob Koskimaki and Prakash Vempati for useful discussions.

## Supporting Information

Text S1 Glossary and system of equations in the absence of luminal receptors and lymphatic drainage
(0.05 MB PDF)
