# Supplementary material for: The Presence of VEGF Receptors on the Luminal Surface of Endothelial Cells Affects VEGF Distribution and VEGF Signaling
Source: PLoS Comput Biol. 2009 Dec 24;5(12):e1000622. doi: 10.1371/journal.pcbi.1000622 (PMC2790341; doi:10.1371/journal.pcbi.1000622)
Supplement: Text S1 — Glossary and system of equations in the absence of luminal receptors and lymphatic drainage (0.05 MB PDF) [file pcbi.1000622.s001.pdf]

Supplemental Information

We present here the chemical reactions as well as the equations provided from [1]. Note that there is no diseased tissue compartment in the present study. The concentrations are noted in brackets (see glossary below).

a. Chemical reactions

$$V_{165} + M_{ECM} \xrightleftharpoons[k_{off,V165,MECM}]{k_{on,V165,MECM}} V_{165}M_{ECM}$$

$$V_{165} + M_{EBM} \xrightleftharpoons[k_{off,V165,MEBM}]{k_{on,V165,MEBM}} V_{165}M_{EBM}$$

$$V_{165} + M_{PBM} \xrightleftharpoons[k_{off,V165,MPBM}]{k_{on,V165,MPBM}} V_{165}M_{PBM}$$

$$V_{165} + R_1 \xrightleftharpoons[k_{off,V165R1}]{k_{on,V165,R1}} V_{165}R_1$$

$$V_{165} + R_2 \xrightleftharpoons[k_{off,V165R2}]{k_{on,V165,R2}} V_{165}R_2$$

$$V_{165} + N_1 \xrightleftharpoons[k_{off,V165N1}]{k_{on,V165,N1}} V_{165}N_1$$

$$V_{165}N_1 + R_2 \xrightleftharpoons[k_{off,V165N1,R2}]{k_{c,V165N1,R2}} R_2V_{165}N_1$$

$$V_{165}R_2 + N_1 \xrightleftharpoons[k_{off,V165R2,N1}]{k_{c,V165R2,N1}} R_2V_{165}N_1$$

$$V_{121} + R_1 \xrightleftharpoons[k_{off,V121R1}]{k_{on,V121,R1}} V_{121}R_1$$

$$V_{121} + R_2 \xrightleftharpoons[k_{off,V121R2}]{k_{on,V121,R2}} V_{121}R_2$$

$$V_{121} + R_1N_1 \xrightleftharpoons[k_{off,V121R1}]{k_{on,V121,R1}} V_{121}R_1N_1$$

$$V_{121}R_1 + N_1 \xrightleftharpoons[k_{dissoc,R1N1}]{k_{c,R1,N1}} V_{121}R_1N_1$$

$$R_1 + N_1 \xrightleftharpoons[k_{dissoc,R1N1}]{k_{c,R1,N1}} R_1N_1$$

b. interstitial space (tissue compartment only)

$$\frac{d\left[M_{EBM}\right]}{dt} = -k_{on,V165,MEBM}\left[V_{165}\right]\left[M_{EBM}\right] + k_{off,V165MEBM}\left[V_{165}M_{EBM}\right] \qquad \text{(S.1)}$$

$$\frac{d\left[M_{ECM}\right]}{dt} = -k_{on,V165,MECM}\left[V_{165}\right]\left[M_{ECM}\right] + k_{off,V165MECM}\left[V_{165}M_{ECM}\right] \qquad \text{(S.2)}$$

$$\frac{d\left[M_{PBM}\right]}{dt} = -k_{on,V165,MPBM}\left[V_{165}\right]\left[M_{PBM}\right] + k_{off,V165MPBM}\left[V_{165}M_{PBM}\right] \qquad \text{(S.3)}$$

$$\frac{d[V_{165}M_{EBM}]}{dt} = k_{on,V165,MEBM}[V_{165}][M_{EBM}] - k_{off,V165MEBM}[V_{165}M_{EBM}] \quad (S.4)$$

$$\frac{d[V_{165}M_{ECM}]}{dt} = k_{on,V165,MECM}[V_{165}][M_{ECM}] - k_{off,V165MECM}[V_{165}M_{ECM}] \quad (S.5)$$

$$\frac{d[V_{165}M_{PBM}]}{dt} = k_{on,V165,MPBM}[V_{165}][M_{PBM}] - k_{off,V165MPBM}[V_{165}M_{PBM}] \quad (S.6)$$

c. cell surface (equations valid for both the tissue and the blood compartments)

$$\begin{aligned}\frac{d[R_1]}{dt} &= s_{R1} - k_{\text{int},R1}[R_1] - k_{on,V165,R1}[V_{165}][R_1] + k_{off,V165R1}[V_{165}R_1] \\ &- k_{on,V121,R1}[V_{121}][R_1] + k_{off,V121R1}[V_{121}R_1] \\ &- k_{c,R1,N1}[N_1][R_1] + k_{dissoc,R1N1}[R_1N_1]\end{aligned} \quad (S.7)$$

$$\begin{aligned}\frac{d[R_2]}{dt} &= s_{R2} - k_{\text{int},R2}[R_2] - k_{on,V121,R2}[V_{121}][R_2] + k_{off,V121R2}[V_{121}R_2] \\ &- k_{on,V165,R2}[V_{165}][R_2] + k_{off,V165R2}[V_{165}R_2] \\ &- k_{c,V165N1,R2}[V_{165}N_1][R_2] + k_{off,V165N1,R2}[R_2V_{165}N_1]\end{aligned} \quad (S.8)$$

$$\begin{aligned}\frac{d[N_1]}{dt} &= s_{N1} - k_{\text{int},N1}[N_1] - k_{c,V121R1,N1}[V_{121}R_1][N_1] + k_{dissoc,R1N1}[V_{121}R_1N_1] \\ &- k_{c,R1,N1}[N_1][R_1] + k_{dissoc,R1N1}[R_1N_1] - k_{on,V165,N1}[V_{165}][N_1] \\ &+ k_{off,V165N1}[V_{165}N_1] - k_{c,V165R2,N1}[V_{165}R_2][N_1] + k_{off,V165R2,N1}[R_2V_{165}N_1]\end{aligned} \quad (S.9)$$

$$\begin{aligned}\frac{d[V_{121}R_1]}{dt} &= -k_{\text{int},V121R1}[V_{121}R_1] + k_{on,V121,R1}[V_{121}][R_1] - k_{off,V121R1}[V_{121}R_1] \\ &- k_{c,R1,N1}[V_{121}R_1][N_1] + k_{dissoc,R1N1}[V_{121}R_1N_1]\end{aligned} \quad (S.10)$$

$$\frac{d[V_{121}R_2]}{dt} = -k_{\text{int},V121R2}[V_{121}R_2] + k_{on,V121,R2}[V_{121}][R_2] - k_{off,V121R2}[V_{121}R_2] \quad (S.11)$$

$$\frac{d[V_{165}R_1]}{dt} = -k_{\text{int},V165R1}[V_{165}R_1] + k_{on,V165,R1}[V_{165}][R_1] - k_{off,V165R1}[V_{165}R_1] \quad (S.12)$$

$$\begin{aligned}\frac{d[V_{165}R_2]}{dt} &= -k_{\text{int},V165R2}[V_{165}R_2] + k_{on,V165,R2}[V_{165}][R_2] - k_{off,V165R2}[V_{165}R_2] \\ &- k_{c,V165R2,N1}[V_{165}R_2][N_1] + k_{off,V165R2,N1}[R_2V_{165}N_1]\end{aligned} \quad (S.13)$$

$$\begin{aligned}\frac{d[V_{165}N_1]}{dt} &= -k_{\text{int},V165N1}[V_{165}N_1] + k_{on,V165,N1}[V_{165}][N_1] - k_{off,V165N1}[V_{165}N_1] \\ &- k_{c,V165N1,R2}[V_{165}N_1][R_2] + k_{off,V165N1,R2}[R_2V_{165}N_1]\end{aligned} \quad (S.14)$$

$$\frac{d\left[R_2V_{165}N_1\right]}{dt} = -k_{\text{int},V165R2N1}\left[R_2V_{165}N_1\right] + k_{c,V165R2,N1}\left[V_{165}R_2\right]\left[N_1\right]$$
$$-k_{off,V165R2,N1}\left[R_2V_{165}N_1\right] + k_{c,V165N1,R2}\left[V_{165}N_1\right]\left[R_2\right] \quad (S.15)$$
$$-k_{off,V165N1,R2}\left[R_2V_{165}N_1\right]$$

$$\frac{d\left[V_{121}R_1N_1\right]}{dt} = -k_{\text{int},V121R1N1}\left[V_{121}R_1N_1\right] + k_{c,V121R1,N1}\left[V_{121}R_1\right]\left[N_1\right]$$
$$-k_{dissoc,V121N1}\left[V_{121}R_1N_1\right] + k_{on,V121,R1N1}\left[V_{121}\right]\left[R_1N_1\right] \quad (S.16)$$
$$-k_{off,V121R1N1}\left[V_{121}R_1N_1\right]$$

$$\frac{d\left[R_1N_1\right]}{dt} = -k_{\text{int},R1N1}\left[R_1N_1\right] + k_{c,R1,N1}\left[N_1\right]\left[R_1\right] - k_{dissoc,R1N1}\left[R_1N_1\right] \quad (S.17)$$
$$-k_{on,V121,R1}\left[V_{121}\right]\left[R_1N_1\right] + k_{off,V121R1}\left[V_{121}R_1N_1\right]$$

d. ligands in the tissue compartment

We denote the tissue compartment by the subscript *N*.

$$\frac{d\left[V_{121}\right]_N}{dt} = q^N_{V121} - k^N_{on,V121,R1}\left[V_{121}\right]_N\left[R_1\right]_N + k^N_{off,V121R1}\left[V_{121}R_1\right]_N$$
$$-k^N_{on,V121,R1N1}\left[V_{121}\right]_N\left[R_1N_1\right]_N + k^N_{off,V121R1N1}\left[V_{121}R_1N_1\right]_N$$
$$-k^N_{on,V121,R2}\left[V_{121}\right]_N\left[R_2\right]_N + k^N_{off,V121R2}\left[V_{121}R_2\right]_N \quad (S.18)$$
$$-k^{NB}_{pV}\frac{S_{NB}}{U_N}\frac{\left[V_{121}\right]_N}{K_{AV,N}} + k^{BN}_{pV}\frac{S_{NB}}{U_N}\frac{U_B}{U_p}\left[V_{121}\right]_B$$

The first term of the equation represents the secretion of the $VEGF_{121}$ isoform by parenchymal cells. The next six terms correspond to the interactions of the $VEGF_{121}$ isoform with its receptors. Finally, the last two terms represent the extravasation and intravasation, respectively, of $VEGF_{121}$. Because of closed pores and inaccessible spaces, free diffusible VEGF is constrained in the "available interstitial fluid volume" $U_{AV} = K_{AV} U$, where K_{AV} is the available volume fraction. The displacement of VEGF molecules from the compartment *i* to *j* follows:

$$U_{AV,i}\frac{d\left[V_{121}\right]_{AV,i}}{dt} = -k^{ij}_{pV}S_{ij}\left[V_{121}\right]_{AV,i} + k^{ji}_{pV}S_{ij}\left[V_{121}\right]_{AV,j} \quad (S.19)$$

which can be re-expressed in terms of $[V_{121}]_j$ by using the relationship $U_{AV,i}\,[V_{121}]_{AV,i} = U_i\,[V_{121}]_i$. Note that, for the blood compartment, $U_p = K_{AV,B}\,U_B$, which means that the volume of plasma is the available fluid volume for VEGF in the blood. Similarly, the equation governing $VEGF_{165}$ is:

$$\frac{d[V_{165}]_N}{dt} = q_{V165}^N - k_{on,V165,MEBM}^N [V_{165}]_N [M_{EBM}]_N + k_{off,V165,MEBM}^N [V_{165}M_{EBM}]_N$$
$$- k_{on,V165,MECM}^N [V_{165}]_N [M_{ECM}]_N + k_{off,V165MECM}^N [V_{165}M_{ECM}]_N$$
$$- k_{on,V165,MPBM}^N [V_{165}]_N [M_{PBM}]_N + k_{off,V165MPBM}^N [V_{165}M_{PBM}]_N \qquad (S.20)$$
$$- k_{on,V165,R1}^N [V_{165}]_N [R_1]_N + k_{off,V165R1}^N [V_{165}R_1]_N - k_{on,V165,R2}^N [V_{165}]_N [R_2]_N$$
$$+ k_{off,V165R2}^N [V_{165}R_2]_N - k_{on,V165,N1}^N [V_{165}]_N [N_1]_N + k_{off,V165N1}^N [V_{165}N_1]_N$$
$$- k_{pV}^{NB} \frac{S_{NB}}{U_N} \frac{[V_{165}]_N}{K_{AV,N}} + k_{pV}^{BN} \frac{S_{NB}}{U_N} \frac{U_B}{U_p} [V_{165}]_B$$

e. ligands in the blood compartment

We denote the blood compartment by the subscript *B*.

$$\frac{d[V_{121}]_B}{dt} = -c_{V121}[V_{121}]_B - k_{pV}^{BN} \frac{S_{NB}}{U_p} [V_{121}]_B + k_{pV}^{NB} \frac{S_{NB}}{U_B} \frac{[V_{121}]_N}{K_{AV,N}} \qquad (S.21)$$
$$\frac{d[V_{165}]_B}{dt} = -c_{V165}[V_{165}]_B - k_{pV}^{BN} \frac{S_{NB}}{U_p} [V_{165}]_B + k_{pV}^{NB} \frac{S_{NB}}{U_B} \frac{[V_{165}]_N}{K_{AV,N}} \qquad (S.22)$$

where c_V represents the clearance of VEGF from the blood.

GLOSSARY

Concentrations and densities	
$[M_{ECM}]$, $[M_{EBM}]$, $[M_{PBM}]$	Density of VEGF binding sites in the ECM, EBM and PBM
$[V_{121}]$, $[V_{165}]$	Concentration of unbound $VEGF_{121}$ and $VEGF_{165}$ in the available interstitial fluid
$[R_1]$, $[R_2]$	Density of the unoccupied receptor tyrosine kinases VEGFR1 and VEGFR2
$[N_1]$	Density of the unoccupied co-receptor (NRP1)
$[R_1N_1]$	Density of the VEGFR1-NRP1 complex
$[V_iR_j]$	Concentration of VEGF isoform *i* bound to VEGF receptor $VEGFR_j$
$[V_iN_1]$	Concentration of VEGF isoform *i* bound to co-receptor NRP1
$[R_2V_{165}N_1]$	Concentration of ternary complex VEGFR2-$VEGF_{165}$-NRP1
$[V_{121}R_1N_1]$	Concentration of ternary complex $VEGF_{121}$-VEGFR1-NRP1
Kinetic parameters	
q_{V121}, q_{V165}	Secretion rate of $VEGF_{121}$ and $VEGF_{165}$
s_R	Rate at which the receptors are inserted into the cell membrane
k_{on}	Kinetic rate for binding

k_c	Kinetic rate for receptor coupling
k_{off}	Kinetic rate for unbinding
k_{int}	Internalization rate of the receptors
k_{pV}^{ij}	Microvascular permeability k_p for VEGF (noted as V) from compartment *i* to *j* (N = tissue; B = blood)
c_{V121}, c_{V165}	Clearance of $VEGF_{121}$ and $VEGF_{165}$ in the blood
Geometric parameters	
U_i	Volume of the compartment *i* (*N* = tissue, *B* = blood, *p* = plasma)
S_{NB}	Total surface of the microvessels at the interface of the tissue (*N*) and the blood (*B*)
$K_{AV,i}$	Available volume fraction in the tissue, i.e., ratio of available fluid volume to total tissue volume U_i

REFERENCE:

1. Stefanini MO, Wu FT, Mac Gabhann F, Popel AS (2008) A compartment model of VEGF distribution in blood, healthy and diseased tissues. BMC Syst Biol 2: 77.
